# Laparoscopic and open postchemotherapy retroperitoneal lymph node dissection in patients with advanced testicular cancer – a single center analysis

**DOI:** 10.1186/1471-2490-12-15

**Published:** 2012-05-31

**Authors:** Jonas Busch, Ahmed Magheli, Barbara Erber, Frank Friedersdorff, Ivan Hoffmann, Carsten Kempkensteffen, Steffen Weikert, Kurt Miller, Mark Schrader, Stefan Hinz

**Affiliations:** 1 From the Charité University Medicine Berlin, Department of Urology, Berlin, Germany

**Keywords:** Advanced testicular cancer, Postchemotherapy, Retroperitoneal lymph node dissection, Laparoscopy, Metastasis

## Abstract

**Background:**

The open approach represents the gold standard for postchemotherapy retroperitoneal lymph node dissection (O-PCLND) in patients with residual testicular cancer. We analyzed laparoscopic postchemotherapy retroperitoneal lymph node dissection (L-PCLND) and O-PCLND at our institution.

**Methods:**

Patients underwent either L-PCLND (n = 43) or O-PCLND (n = 24). Categorical and continuous variables were compared using the Fisher exact test and Mann–Whitney *U* test respectively. Overall survival was evaluated with the log-rank test.

**Results:**

Primary histology was embryonal cell carcinomas (18 patients), pure seminoma (2 cases) and mixed NSGCTs (47 patients). According to the IGCCCG patients were categorized into “good”, “intermediate” and “poor prognosis” disease in 55.2%, 14.9% and 20.8%, respectively. Median operative time for L-PCLND was 212 min and 232 min for O-PCLND (p = 0.256). Median postoperative duration of drainage and hospital stay was shorter after L-PCLND (0.0 vs. 3.5 days; p < 0.001 and 6.0 vs. 11.5 days; p = 0.002). Intraoperative complications occurred in 21.7% (L-PCLND) and 38.0% (O-PCLND) of cases with 19.5% and 28.5% of Clavien Grade III complications for L-PCLND and O-PCLND, respectively (p = 0.224). Significant blood loss (>500 ml) was almost equally distributed (8.6% vs. 14.2%: p = 0.076). No significant differences were observed for injuries of major vessels and postoperative complications (p = 0.758; p = 0.370). Tumor recurrence occurred in 8.6% following L-PCLND and in 14.2% following O-PCLND with a mean disease-free survival of 76.6 and 89.2 months, respectively. Overall survival was 83.3 and 95.0 months for L-PCNLD and O-PCLND, respectively (p = 0.447).

**Conclusions:**

L-PCLND represents a safe surgical option for well selected patients at an experienced center.

## Background

PCLND plays an important role in the management of patients with advanced seminomatous and NSGCT [1-5]. The technical advances in radiographic staging and the increased use of tumor markers have improved the correct identification of candidates for PCLND [6]. However, even with the introduction of FDG-PET for staging of postchemotherapy seminoma patients we cannot reliably rule out viable disease in residual GCT [7]. Postchemotherapy residual masses in patients suffering from non-seminoma should be resected according to large retrospective series and the most recent urological guidelines [6,8]. Traditionally, this resection is performed by an open surgical approach (O-PCLND) - a technically demanding procedure which should be limited to experienced tertiary referral centers [9,10]. The extension of the dissection varies from a radical bilateral approach to a more limited lumpectomy. Full bilateral resection is the standard of care for extensive residual masses. A recent analysis of 152 patients by Heidenreich et al. revealed that in well-defined masses a modified template resection does not worsen oncological outcome, but considerably decreases treatment morbidity [11].

O-PCLND is associated with a significant intra- and postoperative morbidity resulting in prolonged hospital stays frequently. Subramanian and colleagues analyzed 96 O-PCLND patients and reported intraoperative, postoperative and late complications in 12%, 32% and 7% of cases, respectively of which 8% were classified as Clavien Grade III/IV complications [12,13].

The complication rate of primary laparoscopic retroperitoneal lymph node dissection in stage I GCTs varies widely in the literature with incidences of 2.2-20%, the majority of with are vascular injuries [14]. For L-PCLND complication rates of up to 43.8% have been reported, necessitating a high level of laparoscopic training for its prevention [14,15]. At the time of L-PCNLD implementation, smaller series with high conversion rates to open surgery have been reported [16]. However, more recent data restricted to small tumors proved that L-PCLND is feasible and has morbidity rates comparable to O-PCLND [17,18]. So far no study has directly compared intra and postoperative data of L-PCLND patients to a contemporary O-PCLND cohort. Consequently, L-PCLND has not been recommended as a standard surgical approach for patients with residual masses following chemotherapy for advanced testicular cancer [1,4,6].

## Results

### Preoperative patient’ characteristics

A total of 18 patients showed evidence of seminomatous fractions in the primary histology report at the time of orchiectomy (L-PCLND: n = 12; 26.0% and for O-PCLND: n = 6; 28.5%). Two patients of the L-PCLND group had pure seminoma. The most common primary histology was mixed NSGCT (n = 47) with a predominance of ECC in 35 patients (52.2%). There were no statically significant differences with respect to histological subtypes between the two groups except for pure ECC (p = 0.030). Baseline patient characteristics including primary histology, IGCCG prognostic risk scores and number of chemotherapy cycles are displayed in Table 1. Good prognosis patients were more likely to undergo L-PCLND than O-PCLND (63.0% vs. 38.0%; p = 0.021). Ten patients demonstrated S1 tumor marker levels post-chemotherapy. However, all of these patients had a massive decline in marker levels from S3/S2 to S1 and/or a radiological response with significantly decreased tumor extend. A total of 35 patients (52.2%) had clinical stage IIa-IIc disease, while the remaining patients (47.8%) had clinical stage IIIa-IIIc disease. There were no statistically significant differences of the two surgical cohorts with respect to tumor stage (p = 0.586; Table 2).

**Table 1:** Preoperative patients’ characteristics

**Variable**	**L-PCLND**	**O-PCLND**	**p Value**
	**N = 46**	**N = 21**	
histology of orchiectomy; n (%) *^2^			
seminoma*^2^	12 (26.0)	6 (28.5)	1.000 *^1^
pure seminoma	2 (4.3)	0 (0)	1.000 *^1^
teratoma*^2^	9 (19.5)	4 (19.0)	1.000 *^1^
embryonal cell carcinoma	25 (54.3)	10 (47.6)	0.558 *^1^
pure embryonal cell carcinoma	16 (34.7)	2 (9.5)	0.030 *^1^
chorioncarcinoma*^2^	2 (4.3)	4 (19.0)	0.077 *^1^
yolk sack*^2^	7 (15.2)	8 (38.0)	0.055 *^1^
IGCCCG risk score before chemotherapy; n (%)			
good	29 (63.0)	8 (38.1)	0.021 *^1^
intermediate	9 (19.6)	1 (4.8)	
poor	6 (13.0)	8 (38.1)	0.072 *^1^
missing	2 (4.3)	4 (19.0)	
Tumor markers before chemotherapy; n (%)			
Not elevated	5 (10.9)	1 (4.8)	0.065 *^1^
S1	30 (65.2)	8 (38.1)	
S2	5 (10.9)	5 (23.8)	
S3	6 (13.0)	7 (33.3)	
Tumor markers after chemotherapy; n (%)			
Not elevated	38 (82.6)	15 (71.4)	0.545 *^1^
S1	6 (13.0)	4 (19.0)	
missing	2 (4.3)	2 (9.5)	
Cycles of chemotherapy preop; median (range)	3 (3-8)	4 (3-7)	0.005 *^3^
Salvage chemotherapy; n (%)	0 (0)	2 (9.5)	0.095 *^1^

Abbreviations

L-PCLND – laparoscopic postchemotherapy retroperitoneal lymph node dissection; O-PCLND – open postchemotherapy retroperitoneal lymph node dissection; IGCCCG – international germ cell cancer collaboration group; preop – preoperatively (before L-PCLND or O-PCLND); *^1^ – fishers exact test; *^2^ proportion of histologic subtype among others, multiple proportions possible; *^3^ – Man Whitney *U* test.

**Table 2:** Operative characteristics at PCLND

**Variable**	**L-PCLND**	**O-PCLND**	**p Value**
	**N = 46**	**N = 21**	
Clinical stage Lugano; n (%)			
CS IIa	6 (13.0)	1 (4.8)	0.586 *^1^
CS IIb	14 (30.4)	5 (23.8)	
CS IIc	6 (13.0)	3 (14.3)	
CS IIIa	7 (15.2)	2 (9.5)	
CS IIIb	5 (10.9)	2 (9.5)	
CS IIIc	8 (17.4)	8 (38.1)	
Resected template; n (%)			
radical bilateral	12 (26.0)	13 (61.9)	0.002 *^1^
modified template resection*^3^	32 (69.5)	5 (23.8)	
lumpectomy	2 (4.3)	0 (0)	
Median age; yrs (IQR)	32.0 (26.5 – 37.5)	28.0 (22.0 – 34.0)	0.521 *^2^
Median BMI; kg/m^2^ (IQR)	24.0 (21.8 – 26.2)	22.6 (19.3 – 25.8)	0.789 *^2^
Median operative time; min (IQR)	212.0 (145 – 298)	232.5 (181 – 424)	0.256 *^2^
Insertion of wound drainage tube; n (%)	12 (26.0)	15 (71.4)	<0.001 *^1^
Median duration of drainage tube; days (IQR)	0.0 (0.0 – 1.0)	3.5 (2.0 – 6.5)	<0.001 *^2^
Median duration of hospital stay; days (IQR)	6.0 (5.0 – 7.5)	11.5 (7.0 – 16.5)	0.002 *^2^
Intraoperative complications; n (%)			
none	36 (78.3)	13 (61.9)	0.244 *^1^
Clavien I	0 (0)	1 (4.7)	
Clavien II	1 (2.2)	1 (4.7)	
Clavien III	9 (19.6)	6 (28.5)	
Intraoperative conversion rate; n (%)	3 (6.5)	0 (0)	n.a.
Blood loss categories; n (%)			
n.s. < 100 ml	41 (89.1)	15 (71.4)	0.076 *^1^
100-500 ml without transfusion	1 (2.1)	3 (14.2)	
>500 ml or transfusion	4 (8.6)	3 (14.2)	
Intraoperative kidney resection; n (%)	0 (0)	3 (14.3)	0.028 *^1^
Major vascular injuries; n (%)	12 (26.0)	4 (19.0)	0.758 *^1^
Postoperative complications; n (%)*^4^			0.431 *^1^
none	41 (89.1)	18 (85.7)	
Clavien I	4 (8.7)	2 (9.5)	
Clavien III	0 (0)	1 (4.8)	

Abbreviations

L-PCLND – laparoscopic postchemotherapy retroperitoneal lymph node dissection; O-PCLND – open postchemotherapy retroperitoneal lymph node dissection; CS – clinical stage; yrs – years; IQR – interquartile range; n.a. – not applicable; *^1^ – fishers exact test; *^2^ – Mann–Whitney *U* test; *^3^ – modified template resection = nerve sparing technique or unilateral resection; *^4^ – information for one patient in the L-PCLND group was missing.

### Residual tumor characteristics

A detailed analysis of the residual tumor localisation and residual tumor histology is demonstrated in Table 3. Median residual tumors were significantly smaller in L-PCLND compared to O-PCLND (2.2 cm vs. 6.7 cm; p = 0.002) (Table 3).

**Table 3:** Residual tumor characteristics

**Variable**	**L-PCLND**	**O-PCLND**	**p-value**
	**N = 46**	**N = 21**	
Residual tumor localisation; n (%)			
paraaortal	34 (73.9)	13 (61.9)	0.392 *^1^
paracaval	6 (13.0)	4 (19.0)	0.713 *^1^
interaortocaval	10 (21.7)	5 (23.8)	1.000 *^1^
iliacal	5 (10.8)	1 (4.7)	0.654 *^1^
Residual tumor histology; n (%) *^3^			
fibrous scar	28 (60.9)	10 (47.6)	0.426 *^1^
mature teratoma	12 (26.1)	5 (23.8)	1.000 *^1^
vital carcinoma	10 (21.7)	5 (23.8)	1.000 *^1^
missing	1 (2.2)	1 (4.8)	0.532 *^1^
Maximal residual tumor diameter			
Median in cm (IQR)	2.2 (1.5 – 3.9)	6.8 (2.5 – 7.6)	0.002 *^2^

Abbreviations

L-PCLND – laparoscopic postchemotherapy retroperitoneal lymph node dissection; O-PCLND – open postchemotherapy retroperitoneal lymph node dissection; IQR – interquartile range; *^1^ – fishers exact test; *^2^ – Mann–Whitney *U* test; *^3^ – proportion of histologic subtype, multiple proportions in L-PCLND cohort possible.

### Operative characteristics

L-PCLND was performed in 46 patients, whereas 21 patients underwent O-PCLND. Median age at the time of surgery was 32.0 years (range 26.5–37.5) and 28.0 years (range 22.0–34.0), mean BMI 24.0 kg/m^2^ and 22.6 kg/m^2^ for O-PCLND and L-PCLND, respectively (p = 0.521). There were no statistically significant differences with respect to these two variables (p = 0.789). Similarly, the operating time was equivalent (212 vs. 232 min; p = 0.256). Median duration of postoperative drainage (0 vs. 3.5 days; p < 0.001) and median hospital stay (6.0 vs. 11.5 days; p = 0.002) were significantly shorter after L-PCLND.

The extend of retroperitoneal lymph node dissection differed significantly: Radical bilateral lymph node resection was performed in 26.0% and 61.9%, a modified unilateral approach in 69.5% and 23.8% and a simple lumpectomy in 4.3% and 0% of patients undergoing O-PCNLD and L-PCLND, respectively (p = 0.002).

Intraoperative complications occurred in 10 of the L-PCLND and in 8 of the O-PCLND patients (21.7% vs. 38.0%). According to Clavien, the incidence of Grade III intraoperative complications was 19.6% for L-PCLND and 28.5% for O-PCLND (p = 0.244). Due to intraoperative complications, 6.5% of the L-PCLND had to be converted O-PCLND. A significant blood loss >500 ml occurred in 8.6% and 14.2% of cases during L- PCLND O-PCLND, respectively (p = 0.076). A simultaneous nephrectomy was necessary in 3 of the O-PCLND patients (14.3%) while none of the patients received a nephrectomy in the L-PCLND cohort (p = 0.028). Vascular injuries occurred in 26.0% and 19.0% of procedures during L-PCLND and O-PCLND, respectively (p = 0.758). Postoperative complications were observed in 4 L-PCLND and 3 O-PCLND patients (8.6% vs. 14.2%; p = 0.370; Table 2). One O-PCLND patient underwent a relaparotomy due to a suspected small bowel obstruction three days postoperatively. Table 4 depicts a detailed analysis of intraoperative Clavien III complications for both groups. Significant injuries of major vessels (V. cava or aorta) were more frequent in the L-PCLND cohort 26.0 vs. 19.0%, p = 0.758). In univariate analysis, residual tumor size (RR 1.24; CI 1.05-1.47; p = 0.010) and the number of chemotherapy cycles (RR 2.90; CI 1.50-5.63; p = 0.002) were the two only significant predictors of grade III intra and postoperative complications. The surgical approach on the other hand was not associated with intra or postoperative complication rate (p = 0.243).

**Table 4:** Clavien grade III intraoperative complications

**Variable**	**L-PCLND**	**O-PCLND**
	**N = 46**	**N = 21**
Significant lesion of V. cava	4	0
Significant lesion of Aorta	2	1
Significant lesion of iliac artery	1	1
Significant lesion of lumbal venes	1	1
Significant lesion of V. renalis	1	0
Unspecified significant bleeding	1	1
Significant lesion of the ureter	(1)	(1)
Significant lesion of the duodenum	0	2

Abbreviations

L-PCLND – laparoscopic postchemotherapy retroperitoneal lymph node dissection; O-PCLND – open postchemotherapy retroperitoneal lymph node dissection.

### Outcome characteristics

Postinterventionally, a total of 33 L-PCLND patients and 12 O-PCLND patients underwent surveillance with regular follow-up visits; additional chemotherapy cycles were applied to 6.5% and 28.5% of patients following L-PCLND and O-PCLND, respectively (p = 0.020). One patient of the L-PCLND cohort required thoracotomy due to an intrathoracic residual tumor. Three patients were lost to follow-up and detailed follow-up data was not available for nine cases. At a median follow-up of 30.1 (L-PCLND) and 54.5 months (O-PCLND), a total of 7 patients experienced disease recurrence, 8.6% (n = 4) following L-PCLND and 14.2% (n = 3) following O-PCLND. Detailed information on patient characteristics of these seven patients are displayed in Table 5.

**Table 5:** Relapse patients‘characteristics

	***Technique***	***Radical or modified template***	***Relapse localisation (in-field* vs. *out-field)***	***Time to relapse***	***Clinical stage***
Pt.#1	L-PCLND	Modified	Unknown, but massive	5 month	II B
Pt.#2	L-PCLND	Radical bilateral	New intrathoracic and in-field relapse	35 month	III C
Pt.#3	L-PCLND	Modified	New pulmonary mets and in-field relapse	33 month	II C
Pt.#4	L-PCLND	Modified	In-field relapse	4 month	III C
Pt.#5	O-PCLND	Modified	In-field relapse	16 month	III B
Pt.#6	O-PCLND	Radical bilateral	Unknown, diffuse progressive disease	unknown	II B
Pt.#7	O-PCLND	Modified	Unknown, but massive	10 month	III B

Abbreviations:

L-PCLND – laparoscopic postchemotherapy retroperitoneal lymph node dissection; O-PCLND – open postchemotherapy retroperitoneal lymph node dissection; Mo - months.

**Figure 1:**
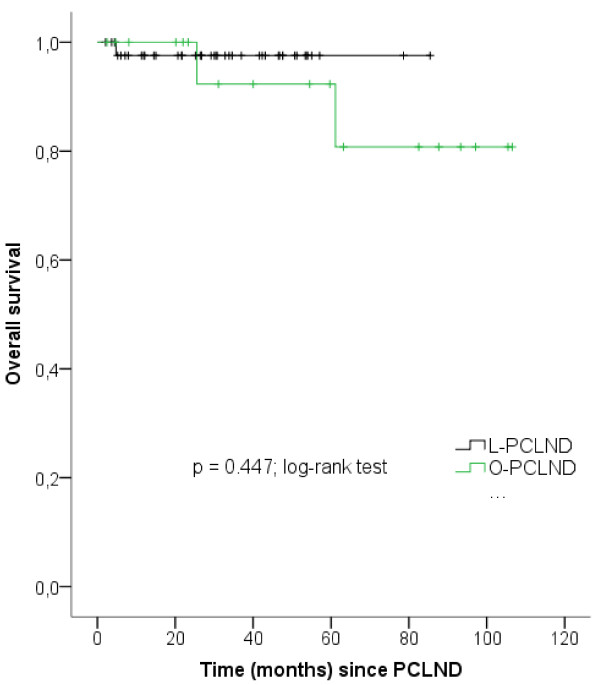
**Mean estimated overall survival since PCLND**. L-PCLND – laparoscopic postchemotherapy retroperitoneal lymph node dissection; O-PCLND – open postchemotherapy retroperitoneal lymph node dissection.

The mean estimated recurrence-free survival was 76.6 months (CI: 68.4-84.8) for L-PCND and 89.2 months (CI: 71.6-106.9) for O-PCLND patients (p = 0.521) with no statistically significant differences between the two cohorts. Similarly, no significant differences in the mean estimated OS were detected (L-PCLND: 83.3 months, CI: 79.6 - 87.3; O-PCLND: 95.0 months, CI: 80.5–109.6; p = 0.447) (Table 6 and Figure 1). In univariate cox regression analysis no predictor of recurrence-free and/or OS was identified. This analysis included surgical technique, IGCCCG risk score, clinical stage and residual tumor diameter (data not shown).

**Table 6:** Outcome characteristics

**Variable**	**L-PCLND**	**O-PCLND**	**p Value**
	**N = 46**	**N = 21**	
Median follow up; months (IQR)	30.1 (12.1 – 47.1)	54.5 (22.0 – 87.7)	0.033 *^1^
Further treatment, n (%)			
surveillance	33 (71.7)	12 (57.1)	0.020 *^2^
chemotherapy	3 (6.5)	6 (28.5)	
thoracotomy	0 (0)	1 (4.8)	
incomplete follow up	9 (19.6)	0 (0)	0.048 *^2^
Lost to follow up	1 (2.2)	2 (9.5)	0.229 *^2^
Tumor relapse, n (%)	4 (8.6)	3 (14.2)	0.415 *^2^
Estimated OS since PCLND in months			
Mean ± SD	83.3 ± 1.9	95.0 ± 7.4	0.447 *^3^
(95%CI; range)	(79.6 - 87.3)	(80.5 – 109.6)	

Abbreviations

L-PCLND – laparoscopic postchemotherapy retroperitoneal lymph node dissection; O-PCLND – open postchemotherapy retroperitoneal lymph node dissection; OS – overall survival; SD – standard deviation; n.s. - not statistically significant; *^1^ – Mann–Whitney *U* test *^2^ – fishers exact test; *^3^ – log-rank test.

## Discussion

In our study comparing O-PCLND and L-PCLND long-term oncological outcome was excellent for both surgical techniques. There were no statistical significant differences between the two cohorts with respect to intraoperative complications. L-PCLND patients were less likely to receive surgical drains. There has been a trend towards a shorter operative time and hospital stay following L-PCLND. To overcome possible confounding factors, like the relatively small number of patients, the retrospective nature of the study and the differences in median residual tumor diameter of the two cohorts, we performed a subgroup analysis of patients with tumor diameters of ≤7 cm. This analysis confirmed the less frequent placement of surgical drains, the shorter duration of drainage and hospital stay. However, despite these efforts, the O-PCLND group was relatively small and the majority of patients had residual tumors larger than 3 cm. Therefore, a sample selection bias might have significantly influenced the results (Table 7). Further confounding factors could be differences in IGCCCG categories and extend of follow-up, the imbalanced distribution of residual tumor diameters and histological subtypes. Therefore, a precise statistical comparison with reliable conclusions remains challenging. Overall this is a descriptive series of patients undergoing PCLND for metastatic testis cancer and some patients were managed in an inhomogeneous fashion. Some of the surgical procedures were not performed according to established treatment guidelines.

**Table 7:** Subgroup analysis of residual tumors ≤7 cm

**Variable**	**L-PCLND**	**O-PCLND**	**p Value**
	**N = 40**	**N = 13**	
Median residual tumor diameter; cm (IQR)	2.1 (1.3 – 3.0)	6.3 (2.5 – 7.0)	0.014*^2^
Insertion of drainage tubes; n (%)	9 (22.5)	10 (76.9)	0.001*^1^
Median time of drainage tube; days (IQR)	0.0 (0.0 – 0.0)	2.5 (0.0 – 4.3)	0.001*^2^
Intraoperative kidney resection; n (%)	0 (0)	1 (7.6)	0.245*^1^
Median operative time in min (IQR)	195.0 (140 – 283)	227.5 (181 – 308)	0.292*^2^
Median time of hospital stay; days (IQR)	5.5 (5.0 – 7.0)	8.5 (5.5 – 13.5)	0.008*^2^

Abbreviations

L-PCLND – laparoscopic postchemotherapy retroperitoneal lymph node dissection; O-PCLND – open postchemotherapy retroperitoneal lymph node dissection; IQR – interquartile range; *^1^ – fishers exact test; *^2^ – Mann–Whitney *U* test.

The results obtained for our L-PCLND patients are comparable to those published previously [17-20]. In 2009 Calestroupat and colleagues reported their experience with 26 L-PCLND patients. Median residual tumor diameter was 3.4 cm, while the conversion rate and the transfusion rate were 11.5% and 3.8%, respectively. Median operative time (183 min) was relatively short with a median hospital stay of 5 days. Grade 3/4 complications occurred in 7.6% of cases. The authors concluded that a high level of surgical expertise is needed to successfully perform a L-PCLND. Although L-PCLND has been restricted to patients with small residual tumors in the study of Calestroupat et al., their conversion rate, transfusion rate and the number of Grade3/4 complication were higher compared to our observations [18].

In a series of 49 L-PCLND patients with residual tumor sizes between 2-5 cm Janetschek and co-workers reported a mean operative time of 226 min and a mean postoperative hospital stay of 3.5 days. Overall, complication rate was low. All bleeding complications were managed laparoscopically and no blood transfusion was necessary. Interestingly, L-PCLND was only applied to patients with clinical stage IIb disease, thereby partly explaining these favourable intra- and postoperative results [17].

The largest L-PCLND series of 59 patients was published by Albqami from Linz, Austria. A mean operative time of 234 min, a conversion rate of 0%, a mean estimated blood loss of 165 ml and a mean hospital stay of 3.8 days were reported. During a 5 year follow-up two patients relapsed [19].

In 2008 Steiner et al. demonstrated the feasibility of a bilateral L-PCLND with the preservation of sympathetic nerves in 42 patients (stage IIB n = 19). No conversion to O-PCLND was necessary; mean operative time was 323 min, no intraoperative complications were reported. Antegrade ejaculation was reported for 85.7% of patients. After a mean follow up of 17.2 months no disease recurrence was observed. Unfortunately, there were no exact tumor diameters and locations reported in this study [20]. Comparison to our data is therefore challenging.

Overall, none of the above mentioned studies directly compared L-PCLND patients with a contemporary O-PCLND cohort.

For O-PCLND patients with initial tumor masses larger than 5 cm local relapse rates of 10% are reported in the literature [21]. However, for extended retroperitoneal teratoma the local disease recurrence rates following surgery might be up to 25% [22]. In a recent series of 73 patients with small residual tumors (mean diameter 4 cm) Luz et al. reported an overall complication rate of 27% and found viable tumor in 22% of patients [23]. In 2007, Carver et al. demonstrated that O-PCLND patients with a residual teratoma had a 10-year recurrence-free survival of 80%. The residual tumor size and the IGCCCG risk classification were independent predictors of disease recurrence [24]. These findings are in contrast to our findings: We did not identify surgical technique, IGCCCG risk score, clinical stage or residual tumor diameter as predictors of disease recurrence or OS. This observation could reflect the significant differences of patient characteristics in Carvers cohort compared to our study, especially with respect to the fraction of L-PCLND patients with good prognosis and small residual tumors.

Subramanian et al. published a detailed analysis of 98 O-PCLND patients. Median blood loss was 1000 ml with a consecutive transfusion rate of 42%. Median operating time was 305 min and the median hospital stay 6 days. Overall, intraoperative complication rate was 12%, grade III complications were reported in 6% of cases, 1% each for grade IV and V. Antegrade ejaculation was preserved in 41% of patients. Unfortunately, similarly to other publications no data on tumor diameters were reported which limits comparability to our data [13].

Reviewing O-PCLND series Heidenreich and co-workers concluded that in advanced NSGCTs, a complete resection should be performed for all residual masses irrespective of tumor size, location or histology, thereby providing an excellent long-term disease-free survival of 95% [9]. For smaller tumors, a modified template resection was recommended [11].

Despite the advances in laparoscopic surgery at highly specialized urological centers, L-PCLND still represents an evolving technique. Patient counselling and decision making on surgical technique used largely depends on the surgeon’s experience, tumor characteristics and the patient’s condition.

One of the major drawbacks in obtaining evidence on this important issue is the fact that comparison of the data published in the literature is challenging due to a number of reasons: firstly different eras, in which interventions were performed [25] and secondly different reporting systems of complications. To overcome these issues partly, we incorporated the recently introduced Clavien classification into our study [12]. Unfortunately, most of the previous studies did not use this evidence based classification, thereby precluding a sufficient comparison.

## Conclusions

To our knowledge this is the largest study directly comparing L-PCLND with O-PCLND patients. In concordance with previous studies we demonstrated that L-PCLND to represents a safe and reasonable alternative option to O-PCLND for well selected patients with small residual tumors. Both approaches are technically demanding and require a high degree of surgical expertise.

Larger multicenter studies are urgently needed adjust for confounders and better define factors qualifying patients for L-PCLND.

## Methods

All patients underwent either L-PCLND (n = 46) or O-PCLND (n = 21) at the Charité University Medicine Berlin between October 1999 and March 2010. The Charité department of urology is performing surgery in two different locations. On one campus all residual tumor resections were performed laparoscopically on the other campus all by the open approach. Therefore open and laparoscopic cases were performed concurrently and consecutively.

Data collection was accomplished under an internal review board-approved protocol via retrospective chart review by means of a standardized protocol of the GTCSG. Follow-up was obtained by phone interview with the patients and their local urologists.

Variables of interest were preoperative data on primary histology at the time of orchiectomy, the IGCCCG prognostic risk score at initial chemotherapy as well as tumor marker levels prior to and after chemotherapy [6]. Additionally, patient characteristics and operative details (operative technique, operative time, duration of surgical drainage, intra- and postoperative complications according to Clavien [26], categorized blood loss, concomitant nephrectomy or injury of major vessels) were analyzed.

L-PCLND and O-PCLND technique is described elsewhere [18,27]. In both surgical approaches a modified template resection was defined by the anatomic boundaries described by Heidenreich and Lattouf et al. [11,27]. Briefly, the right-sided modified template resection consisted of the precaval, paracaval, retrocaval, and interaortocaval regions as well as the area lateral to the common iliac vessels with the crossing of the ureter as a caudal boundary. The ureter served as the lateral and the renal vein as the cranial boundary. Similarly the left-sided modified template resection included the preaortic area down to the inferior mesenteric artery, the para-aortic and retroaortic regions with the ureteral crossing of the iliac artery as the caudal and the ureter as the lateral boundaries of dissection. A radical template resection was performed in case of contralateral spread, interaortocaval tumor location, or larger residual tumors. For a radical bilateral approach, the dissection fields of the right- and left-modified resections were combined. In defined cases, a nerve-sparing technique as described by Steiner et al. was performed and considered a modified approach [20]. Lumpectomy was defined as a simple resection of the residual tumor.

Blood loss category I was defined as insignificant if <100 ml without transfusion, category II if between 100 ml and 500 ml without transfusion and category III if blood loss was >500 ml with or without the need for intraoperative blood transfusions. The rationale for using surgical drains in the L-PCLND cohort largely depended on the surgeon’s intraoperative assessment of wound secretion.

Differences in categorical variables were analyzed by the Fisher’s exact test, while continuous variables were analysed using the Mann–Whitney *U* test for non-normally distributed data. Differences in mean estimated OS were calculated by the log-rank test. Predictors of disease-free survival and OS were analyzed in a univariate cox regression model. All tests were two-tailed, differences were considered significant with a p value <0.05. The statistical analysis was conducted with SPSS version 18.0 (SPSS Inc., Chicago, IL, USA).

## Abbreviations

PCLND: postchemotherapy retroperitoneal lymph node dissection; O-PCLND: open PCLND; L-PCLND: laparoscopic PCLND; FDG-PET: FDG-positron emission tomography; GTCSG: German Testicular Cancer Study Group; GCT: germ cell tumor; NSGCT: non-seminomatous germ cell tumor; OS: overall survival; CI: confidence interval; TIP: etoposid ifosfamide, cis-platin; IGCCCG: International Germ Cell Consensus Conference Group; ECC: embryonal cell carcinoma; BMI: body mass index; RR: relative risk.

## Competing interests

The authors declare that they have no competing interests.

## Authors’ contributions

Conception and design: JB, KM, MS, SH Acquisition of data: JB, IH, BE, FF, AM Analysis and interpretation of the data: JB, BE, SH Drafting of the manuscript: JB, SH Critical review of the manuscript for important intellectual content: JB, IH, BE, FF, CK, SH, AM, KM, SW, MS Statistical analysis: JB, BE, SH Administrative, technical or material support: SW, MS, SH, KM, CK Supervision: AM, SW, KM, CK, MS. All authors read and approved the final manuscript.

## Pre-publication history

The pre-publication history for this paper can be accessed here:

http://www.biomedcentral.com/1471-2490/12/15/prepub

## References

[B1] Schmoll HJ Jordan K Huddart R Laguna MP Horwich A Fizazi K Kataja V Testicular non-seminoma: ESMO clinical recommendations for diagnosis, treatment and follow-up Ann Oncol 2009 20 Suppl 4 89 96 19454475 10.1093/annonc/mdp139

[B2] Schmoll HJ Jordan K Huddart R Laguna MP Horwich A Fizazi K Kataja V Testicular seminoma: ESMO clinical recommendations for diagnosis, treatment and follow-up Ann Oncol 2009 20 Suppl 4 83 88 19454474 10.1093/annonc/mdp138

[B3] Krege S Beyer J Souchon R Albers P Albrecht W Algaba F Bamberg M Bodrogi I Bokemeyer C Cavallin-Stahl E European consensus conference on diagnosis and treatment of germ cell cancer: a report of the second meeting of the European Germ Cell Cancer Consensus group (EGCCCG): part I Eur Urol 2008 53 3 478 496 10.1016/j.eururo.2007.12.024 18191324

[B4] Krege S Beyer J Souchon R Albers P Albrecht W Algaba F Bamberg M Bodrogi I Bokemeyer C Cavallin-Stahl E European consensus conference on diagnosis and treatment of germ cell cancer: a report of the second meeting of the European Germ Cell Cancer Consensus Group (EGCCCG): part II Eur Urol 2008 53 3 497 513 10.1016/j.eururo.2007.12.025 18191015

[B5] Einhorn LH Donohue JP Advanced testicular cancer: update for urologists J Urol 1998 160 6 Pt 1 1964 1969 9817300

[B6] Albers P Albrecht W Algaba F Bokemeyer C Cohn-Cedermark G Fizazi K Horwich A Laguna MP EAU Guidelines on Testicular Cancer: 2011 Update Eur Urol 2011 60 2 304 319 10.1016/j.eururo.2011.05.038 21632173

[B7] Hinz S Schrader M Kempkensteffen C Bares R Brenner W Krege S Franzius C Kliesch S Heicappel R Miller K The role of positron emission tomography in the evaluation of residual masses after chemotherapy for advanced stage seminoma J Urol 2008 179 3 936 940 discussion 940 10.1016/j.juro.2007.10.054 18207171

[B8] Albers P Weissbach L Krege S Kliesch S Hartmann M Heidenreich A Walz P Kuczyk M Fimmers R Prediction of necrosis after chemotherapy of advanced germ cell tumors: results of a prospective multicenter trial of the German Testicular Cancer Study Group J Urol 2004 171 5 1835 1838 10.1097/01.ju.0000119121.36427.09 15076288

[B9] Heidenreich A Thuer D Polyakov S Postchemotherapy retroperitoneal lymph node dissection in advanced germ cell tumours of the testis Eur Urol 2008 53 2 260 272 10.1016/j.eururo.2007.10.033 18045770

[B10] Capitanio U Jeldres C Perrotte P Isbarn H Crepel M Cloutier V Baillargeon-Gagne S Shariat SF Duclos A Arjane P Population-based study of perioperative mortality after retroperitoneal lymphadenectomy for nonseminomatous testicular germ cell tumors Urology 2009 74 2 373 377 10.1016/j.urology.2009.01.085 19501893

[B11] Heidenreich A Pfister D Witthuhn R Thuer D Albers P Postchemotherapy retroperitoneal lymph node dissection in advanced testicular cancer: radical or modified template resection Eur Urol 2009 55 1 217 224 10.1016/j.eururo.2008.09.027 18926622

[B12] Clavien PA Barkun J de Oliveira ML Vauthey JN Dindo D Schulick RD de Santibanes E Pekolj J Slankamenac K Bassi C The Clavien-Dindo classification of surgical complications: five-year experience Ann Surg 2009 250 2 187 196 10.1097/SLA.0b013e3181b13ca2 19638912

[B13] Subramanian VS Nguyen CT Stephenson AJ Klein EA Complications of open primary and post-chemotherapy retroperitoneal lymph node dissection for testicular cancer Urol Oncol 2008 28 5 504 509 19097812 10.1016/j.urolonc.2008.10.026

[B14] Kenney PA Tuerk IA Complications of laparoscopic retroperitoneal lymph node dissection in testicular cancer World J Urol 2008 26 6 561 569 10.1007/s00345-008-0299-3 18594824

[B15] Permpongkosol S Lima GC Warlick CA Allaf ME Varkarakis IM Bagga HS Kohanim S Kavoussi LR Postchemotherapy laparoscopic retroperitoneal lymph node dissection: evaluation of complications Urology 2007 69 2 361 365 10.1016/j.urology.2006.10.020 17320678

[B16] Rassweiler JJ Henkel TO Stock C Seemann O Frede T Alken P Retroperitoneal laparoscopic lymph node dissection for staging non-seminomatous germ cell tumors before and after chemotherapy Lymphology 1996 29 1 36 44 8721978

[B17] Janetschek G Peschel R Hobisch A Bartsch G Laparoscopic retroperitoneal lymph node dissection J Endourol 2001 15 4 449 453 discussion 453-445 10.1089/089277901300189538 11394460

[B18] Calestroupat JP Sanchez-Salas R Cathelineau X Rozet F Galiano M Smyth G Kasraeian A Barret E Vallancien G Postchemotherapy laparoscopic retroperitoneal lymph node dissection in nonseminomatous germ-cell tumor J Endourol 2009 23 4 645 650 10.1089/end.2008.0423 19335332

[B19] Albqami N Janetschek G Laparoscopic retroperitoneal lymph-node dissection in the management of clinical stage I and II testicular cancer J Endourol 2005 19 6 683 692 discussion 692 10.1089/end.2005.19.683 16053357

[B20] Steiner H Zangerl F Stohr B Granig T Ho H Bartsch G Peschel R Results of bilateral nerve sparing laparoscopic retroperitoneal lymph node dissection for testicular cancer J Urol 2008 180 4 1348 1352 discussion 1352-1343 10.1016/j.juro.2008.06.040 18707723

[B21] Flechon A Tavernier E Boyle H Meeus P Rivoire M Droz JP Long-term oncological outcome after post-chemotherapy retroperitoneal lymph node dissection in men with metastatic nonseminomatous germ cell tumour BJU Int 2010 106 6 779 785 10.1111/j.1464-410X.2009.09175.x 20089110

[B22] Beck SD Foster RS Bihrle R Einhorn LH Donohue JP Long-term outcome for patients with high volume retroperitoneal teratoma undergoing post-chemotherapy surgery J Urol 2009 181 6 2526 2532 10.1016/j.juro.2009.01.116 19371895

[B23] Luz MA Kotb AF Aldousari S Brimo F Tanguay S Kassouf W Aprikian AG Retroperitoneal lymph node dissection for residual masses after chemotherapy in nonseminomatous germ cell testicular tumor World J Surg Oncol 2010 8 97 10.1186/1477-7819-8-97 21062470 PMC2991320

[B24] Carver BS Shayegan B Serio A Motzer RJ Bosl GJ Sheinfeld J Long-term clinical outcome after postchemotherapy retroperitoneal lymph node dissection in men with residual teratoma J Clin Oncol 2007 25 9 1033 1037 10.1200/JCO.2005.05.4791 17261854

[B25] Mosharafa AA Foster RS Koch MO Bihrle R Donohue JP Complications of post-chemotherapy retroperitoneal lymph node dissection for testis cancer J Urol 2004 171 5 1839 1841 10.1097/01.ju.0000120141.89737.90 15076289

[B26] Dindo D Demartines N Clavien PA Classification of surgical complications: a new proposal with evaluation in a cohort of 6336 patients and results of a survey Ann Surg 2004 240 2 205 213 10.1097/01.sla.0000133083.54934.ae 15273542 PMC1360123

[B27] Lattouf JB Jeschke S Janetschek G Laparoscopic retroperitoneal lymph node dissection: technique BJU Int 2007 100 6 1415 1429 10.1111/j.1464-410X.2007.07297.x 17979941

